# Cardiometabolic index is associated with increased bone mineral density: a population-based cross-sectional study

**DOI:** 10.3389/fpubh.2024.1403450

**Published:** 2024-09-18

**Authors:** Xujin Wu, Xixin Jin, Wei Xu, Chang She, Liubing Li, Yongtao Mao

**Affiliations:** Department of Orthopedics, The Second Affiliated Hospital of Soochow University, Suzhou, China

**Keywords:** CMI, BMD, osteoporosis, NHANES, cross-sectional study

## Abstract

**Background:**

Osteoporosis is a multifactorial bone disease in which lipid metabolism plays an important role. Bone Mineral Density (BMD) measured by Dual-energy X-ray Absorptiometry (DXA) is a critical indicator for diagnosing osteoporosis. The cardiometabolic index (CMI) is a novel metric that combines two quantitative indicators of blood lipids—triglycerides (TG) and high-density lipoprotein cholesterol (HDL-C). This study explores the association between CMI and BMD and seeks to elucidate the role of lipid metabolism in the context of bone health.

**Methods:**

Based on the data of the National Health and Nutrition Examination Survey (NHANES) 2017–March 2020-pre-pandemic, weighted multiple linear regression and smooth curve fitting were used to study the relationship between CMI and femoral BMD. Stratified analyses were also conducted for age, gender, BMI, ethnicity, diabetes and hypertension status. And, the saturation threshold effect of CMI was further analyzed using a two-stage linear regression model.

**Result:**

This study enrolled a total of 1,650 participants (48.7% males), with an average age of 63.0 ± 8.6 years. After adjusting for multiple confounding factors, CMI was positively correlated with total femur BMD, trochanter BMD, and intertrochanter BMD, while the correlation with femur neck BMD was not statistically significant. In the fully adjusted model, each unit increase in CMI was associated with a 0.026 (g/cm^2^) increase in total femur BMD, a 0.022 (g/cm^2^) increase in trochanter BMD, and a 0.034 (g/cm^2^) increase in intertrochanter BMD. Subjects in the highest quartile of CMI had a 0.034 (g/cm^2^) increase in total femur BMD, a 0.035 (g/cm^2^) increase in trochanter BMD, and a 0.039 (g/cm^2^) increase in intertrochanter BMD in the fully-adjusted model compared to those in the lowest quartile. In addition, saturation was observed between CMI and total femur BMD, trochanter BMD and intertrochanter BMD, with saturation thresholds of 1.073, 1.431 and 1.073, respectively.

**Conclusion:**

CMI is strongly associated with BMD, indicating its potential relevance in bone metabolism. However, the role of CMI in the context of bone health, especially regarding osteoporosis risk, requires further investigation in large-scale prospective studies.

## Introduction

Osteoporosis (OP) is a systemic skeletal disease characterized by an increased risk of fractures (1). Approximately 200 million people worldwide are affected by osteoporosis, and according to NHANES data, more than 16.20% of the population in the United States suffers from osteoporosis, and as aging intensifies, this proportion will gradually increase (2, 3). Osteoporosis is characterized by a loss of bone mass and damage to the microstructure of the skeleton, significantly increases the incidence of fragility fractures (4, 5). Femoral BMD is an important indicator for the detection and diagnosis of osteoporosis. It is strongly associated with all-cause mortality in osteoporosis patients, with hip fractures due to decreased femoral BMD being even more devastating in the older adult population (6, 7). The World Health Organization (WHO) has proposed that Bone Mineral Density (BMD) measured by Dual Energy X-ray Absorptiometry (DXA) is the gold standard for diagnosing osteoporosis, which helps identify potential risk factors for bone health and plays a crucial role in the prevention and early detection of osteoporosis (8).

Recent research has revealed a close relationship between lipid metabolism and bone metabolism. Specifically, studies shown that osteoporosis is strongly associated with high-density lipoprotein cholesterol (HDL-C) and triglycerides (TG) (9, 10). Moreover, Gender differences significantly influence osteoporosis development and progression. Postmenopausal women are at heightened risk due to hormonal changes, particularly estrogen deficiency, which accelerates bone loss and adversely affects lipid metabolism, increasing osteoporosis risk (11, 12). However, there is a lack of consistent evidence on the relationship between lipid metabolism and osteoporosis.

Recent studies have observed increased bone mineral density and altered bone health in patients with high-risk cardiometabolic conditions, such as prediabetes, type 2 diabetes, and non-alcoholic fatty liver disease, suggesting a potential link between cardiometabolic risk factors and bone health (13–15). The cardiometabolic index (CMI) has been recognized as a new indicator of the distribution and dysfunction of visceral adipose tissue and primarily used to assess cardiovascular disease risk. CMI not only indicates an individual’s degree of obesity but also reflects blood lipid levels (16, 17). However, its correlation with osteoporosis and BMD remains unclear. Therefore, this study used the National Health and Nutrition Examination Survey (NANES) database to examine the relationship between CMI and BMD at different sites in the femur, thereby investigating the link between lipid metabolism and osteoporosis.

## Materials and methods

### Reach publication

The NHANES database is the largest population-based national nutritional health survey in the world, managed by the U.S. National Center for Health Statistics (NCHS). The survey has been conducted biennially since 1999, utilizing a complex, stratified, multistage sampling design to select representative populations. More detailed information about the NHANES database can be found on the NHANES website: http://www.cdc.gov/nchs/nhanes/.

This study focuses on the 2017–2020-pre-pandemic NHANES database, which includes 15,560 participants, and aims to assess the nutritional and health status of Americans. Among these participants, 3,445 underwent DXA testing. After excluding subjects with missing relevant covariates, the study included a total of 1,650 subjects. The survey collected data through household questionnaires, telephone interviews, and examinations conducted by medical professionals and trained staff. Further details can be found at https://www.cdc.gov/nchs/nhanes/irba98.htm.

### Variable

The independent variable in this study was CMI, which was derived from anthropometric indicators and blood samples. Data were collected based on standardized sampling protocols and rigorous laboratory tests and measurements to ensure validity and accuracy. Blood samples were typically collected in an investigative vehicle or at a designated sampling site and then processed and tested in a standard laboratory. Subjects’ height and waist circumference were measured by certified health professionals in a mobile screening facility. Based on the above indicators, calculate WHtR and CMI:

WHtR=waist circumference(cm)/height(cm) ;

CMI=TG(mmol/L)/HDL−C(mmol/L)×WHtR .

The dependent variable was BMD measured by NHANES DXA using a Hologic Discovery Model A densitometer with APEX 3.2. BMD (g/cm^2^) was defined as bone mineral content (g) divided by bone area (cm^2^). Specific data on BMD measurements using DXA can be found on the website: https://wwwn.cdc.gov/nchs/nhanes/Search/DataPage.aspx?Component=Examination, particularly in the chapter “Dual-Energy X-ray Absorptiometry—Whole Body.”

Based on previous studies, confounders that could potentially affect BMD were selected to eliminate potential effects on outcomes (18, 19). We also analyzed these covariates for multicollinearity and no multicollinearity was detected. Finally, the following covariates were collected and adjusted for gender, age, race, PIR (Poverty Income Ratio), body mass index (BMI), blood urea nitrogen (mg/dl), creatinine (mg/dl), globulin (g/dl), total protein (g/dl), uric acid (mg/dl), glycohemoglobin (%), low-density lipoprotein cholesterol (mmol/L), serum phosphorus (mmol/l), serum iron (μmol/l), serum calcium (mmol/l), and smoking status, alcohol consumption, and the presence of hypertension or diabetes. For more information on covariates, see the NHANES website: http://www.cdc.gov/nchs/nhanes/.

### Data analysis

Continuous variables were expressed as mean values with standard deviations (mean ± SD), while categorical variables were presented as percentages. The comparison of continuous variables was conducted using a weighted t-test, and for categorical variables, a chi-squared test was applied, with outcomes reported as counts (n) and percentages (%). Multivariate regression models were used to assess the relationship between CMI and BMD. To assess the correlation between covariates and the analytical outcomes, three distinct models were formulated. Each model in the analysis progressively incorporated additional adjustments for covariates. The initial model remained unadjusted, while the second model included partial adjustments for age, gender and race. Model 3 represents the fully adjusted model, encompassing additional variables such as BMI, smoking habits, alcohol consumption, diabetes, hypertension and so on. Following this, subgroup analyses were executed to explore potential modifications in effect measures, including gender, ethnicity, age and BMI as potential influential factors. Finally, the saturation threshold effect of CMI was further analyzed using a two-stage linear regression model. Statistical analyses were conducted using R and Empower Stats, with significance set at *p* < 0.05 and strong significance at *p* < 0.01.

### Ethics approval and consent to participate

NHANES participants were required to sign an informed consent form, and the data are now publicly available. The study was reviewed and approved by the Research Ethics Review Board of the National Center for Health Statistics (NCHS). The acquisition and dissemination of data within the NHANES database adhered to the principles of the Declaration of Helsinki, with the necessary approval from the Ethics Committee to ensure the ethical integrity of the data used in this investigation. The research methodology is based entirely on publicly available statistical data. All research activities complied with applicable laws and ethical standards in accordance with the guidelines for data usage and research practices.

## Result

### Baseline characteristic

A total of 1,650 subjects were included in this study, mean age: 63.0 ± 8.6 year, of which 48.7% were male and 51.3% female. Subjects included 5.1% Mexican American, 6.8% Other Hispanic, 69.8% Non-Hispanic White people, 9.3% Non-Hispanic Black people, 5.8% Non-Hispanic Asian and 3.3% Other Race—Including Multi-Racial.

As shown in Table 1, subjects were categorized into CMI quartiles: Q1 (0.041–0.283), Q2 (0.284–0.485), Q3 (0.486–0.831), and Q4 (0.832–24.483), based on the 25th, 50th, and 75th percentiles of the CMI distribution. Most of the covariates in each subgroup were significantly different from each other. We found that populations with higher CMI (Q4) were predominantly male, non-Hispanic white people, hypertensive, and non-diabetic. And with increasing CMI, there was a gradual increase in BMI, hip circumference, blood urea nitrogen, globulin, blood uric acid, glycated glycohemoglobin, total cholesterol, low-density lipoprotein cholesterol (*p* < 0.001), and a gradual increase in bone mineral density of the femur at all sites of the femur (*p* < 0.001) in the population. On the contrary, Serum iron gradually decreased with increasing CMI (*p* < 0.001).

**Table 1:** Weighted characteristics of the study population based on CMI.

Characteristic	Q1: 0.041–0.283	Q2: 0.284–0.485	Q3: 0.486–0.831	Q4: 0.832–24.483	*p*-value
Age (year)	63.2 ± 9.1	63.4 ± 8.3	63.1 ± 8.7	62.5 ± 8.2	0.466
Gender (%)					<0.001
Male	39.3	46.5	47.6	61.1	
Female	60.7	53.5	52.4	38.9	
Race (%)					<0.001
Mexican American	2.3	5.0	6.8	6.7	
Other Hispanic	5.1	7.2	7.5	7.4	
Non-Hispanic White	69.3	69.3	64.8	75.0	
Non-Hispanic Black	13.1	10.6	8.7	4.6	
Non-Hispanic Asian	6.1	5.5	7.8	3.9	
Other Race-Including Multi-Racial	4.2	2.4	4.3	2.3	
BMI	25.9 ± 4.6	27.8 ± 4.8	30.5 ± 5.6	32.4 + 6.3	<0.001
Hip Circumference (cm)	101.3 ± 9.6	104.5 ± 11.0	108.9 ± 12.5	111.4 ± 14.0	<0.001
Poverty income ratio	3.4 ± 1.6	3.5 ± 1.6	3.3 ± 1.7	3.2 ± 1.6	0.046
Alcohol drinking in the past 12 months (%)					0.368
Yes	92.4	89.1	91.9	91.3	
No	7.4	10.9	8.1	8.7	
Smoke ≥100 cigarettes in life					0.001
Yes	43.8	56.1	55.7	45.0	
No	56.2	43.9	44.3	55.0	
Hypertension (%)					0.890
Yes	29.4	29.9	31.8	30.4	
No	70.6	70.1	68.2	69.6	
Diabetes (%)					<0.001
Yes	4.3	11.8	21.2	33.6	
No	95.7	88.2	78.8	66.4	
Blood Urea Nitrogen (mmol/L)	5.784 ± 1.955	5.722 ± 2.040	5.638 ± 1.814	6.329 ± 2.278	<0.001
Blood Creatinine (mg/dl)	0.878 ± 0.298	0.912 ± 0.354	0.884 ± 0.221	0.939 ± 0.457	0.045
Globulin (g/dL)	2.918 ± 0.423	2.997 ± 0.438	3.025 ± 0.428	3.056 ± 0.393	<0.001
Blood Total Protein (g/dl)	69.821 ± 4.708	70.122 ± 4.079	70.472 ± 4.419	70.512 ± 4.120	0.0741
Uric Acid (mg/dl)	4.846 ± 1.222	5.331 ± 1.352	5.847 ± 1.379	6.029 ± 1.411	<0.001
Glycohemoglobin (%)	5.575 ± 0.600	5.775 ± 0.677	6.004 ± 1.013	6.322 ± 1.292	<0.001
Serum phosphorus (mmol/dl)	1.147 ± 0.156	1.118 ± 0.171	1.129 ± 0.167	1.118 ± 0.165	0.032
Serum calcium (mmol/l)	2.319 ± 0.086	2.317 ± 0.084	2.326 ± 0.104	2.315 ± 0.090	0.336
Serum iron (ug/dl)	16.655 ± 6.292	17.577 ± 5.807	17.816 ± 6.608	16.358 ± 4.893	<0.001
HDL-C (mmol/L)	1.878 ± 0.428	1.521 ± 0.281	1.302 ± 0.240	1.076 ± 0.187	<0.001
TC (mmol/L)	4.915 ± 0.934	4.952 ± 1.002	4.848 ± 1.135	5.088 ± 1.257	0.015
TG (mmol)	0.636 ± 0.178	0.989 ± 0.225	1.330 ± 0.288	2.293 ± 1.788	<0.001
LDL-C (mmol/L)	2.745 ± 0.800	2.979 ± 0.879	2.937 ± 1.019	3.021 ± 1.113	<0.001
WHtR	0.560 ± 0.071	0.591 ± 0.072	0.630 ± 0.081	0.660 ± 0.086	<0.001
Total femur BMD (g/cm^2^)	0.867 ± 0.145	0.908 ± 0.156	0.945 ± 0.145	0.987 ± 0.144	<0.001
Femur neck BMD (g/cm2)	0.729 ± 0.145	0.751 ± 0.145	0.776 ± 0.133	0.803 ± 0.130	<0.001
Trochanter BMD (g/cm^2^)	0.648 ± 0.118	0.690 ± 0.126	0.710 ± 0.122	0.747 ± 0.122	<0.001
Intertrochanter BMD (g/cm^2^)	1.032 ± 0.175	1.078 ± 0.186	1.128 ± 0.168	1.174 ± 0.175	<0.001
CMI	0.196 ± 0.059	0.382 ± 0.056	0.642 ± 0.097	1.470 ± 1.517	<0.001

Mean ± SD for continuous variables: *p*-value was calculated by weighted linear regression model. (%) for categorical variables: *p*-value was calculated by weighted chi-square test. CMI: Cardiometabolic index; BMI: body mass index; TC: total cholesterol, TG: triglyceride; LDL-C: low-density lipoprotein cholesterol; HDL-C: high-density lipoprotein cholesterol; WHtR: waist circumference (cm)/height (cm); BMD: bone mineral density.

### Association between CMI and BMD

**Figure 1:**
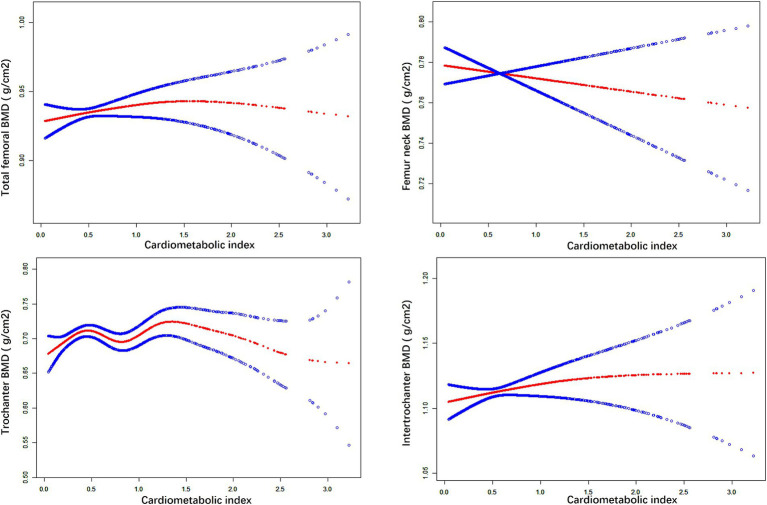
The nonlinear associations between CMI and BMD. The solid red line represents the smooth curve fit between variables. Blue bands represent the 95% of confidence interval from the fit.

We used weighted multiple linear regression models to reveal the relationship between CMI and BMD. As shown in Table 2, in the simple adjustment model (Model2), there is a significant positive correlation between CMI and femoral BMD: total femoral BMD (0.019 (0.012, 0.025) <0.001), Femur neck BMD (0.009 (0.003, 0.016) 0.006), trochanter BMD (0.015 (0.009, 0.021) <0.001), intertrochanter BMD (0.022 (0.014, 0.030) <0.001). In the fully adjusted model (Model3), the positive correlation between CMI and femur neck BMD was not significant (0.001 (−0.014, 0.016) 0.904) but remained significant with total femur BMD (0.026 (0.010, 0.041) 0.001), trochanter BMD (0.022 (0.008, 0.035) 0.002) and intertrochanter BMD (0.034 (0.015, 0.053) <0.001) remained significantly positively correlated. It can be found that in the fully adjusted model (Model3), for each 1-unit increase in CMI, the corresponding increase in total femoral BMD was 0.026 g/cm^2^, trochanter BMD was 0.015 g/cm^2^, and intertrochanter BMD was 0.022 g/cm^2^. When CMI was grouped according to quartiles using CMI Q1 as the reference, in the fully adjusted model (model3), the contribution of CMI to total femoral BMD (0.034 (0.013, 0.055) 0.001), trochanter BMD (0.035 (0.017, 0.054) <0.001) and intertrochanter BMD (0.039 (0.014, 0.065) 0.003) remained significant and the trend between each group is also statistically different (*p* for trend <0.05). In addition, the results of smoothed curve fitting (Figure 1) further demonstrated the positive correlation of CMI on total femoral BMD, trochanter BMD, and intertrochanter BMD, but the effect of CMI on femur neck BMD was not significant. Interestingly, as CMI increased, there was a brief period of decline in trochanter BMD followed by an upward trend.

**Table 2:** Associations between cardiometabolic index and BMD.

	Exposure	Model 1 β (95% CI), *p* value	Model 2 β (95% CI), *p* value	Model 3 β (95% CI), *p* value
Total femur BMD	CMI	0.028 (0.020, 0.036) <0.001	0.019 (0.012, 0.025) <0.001	0.026 (0.010, 0.041) 0.0012
	CMI Q1	Reference	Reference	Reference
	CMI Q2	0.041 (0.021, 0.061) <0.001	0.033 (0.016, 0.050)<0.001	0.014 (−0.003, 0.032) 0.111
	CMI Q3	0.077 (0.057, 0.098) <0.001	0.068 (0.050, 0.086) <0.001	0.020 (0.000, 0.040) 0.045
	CMI Q4	0.120 (0.100, 0.140) <0.001	0.092 (0.075, 0.110) <0.001	0.034 (0.013, 0.055) 0.001
	P for trend	<0.001	<0.001	0.002
Femur neck BMD	CMI	0.015 (0.008, 0.022) <0.001	0.009 (0.003, 0.016) 0.006	0.001 (−0.014, 0.016) 0.904
	CMI Q1	Reference	Reference	Reference
	CMI Q2	0.023 (0.004, 0.041) 0.01481	0.019 (0.003, 0.035) 0.023	0.002 (−0.015, 0.019) 0.799
	CMI Q3	0.047 (0.028, 0.067) <0.001	0.044 (0.027, 0.061) <0.001	0.003 (−0.016, 0.023) 0.742
	CMI Q4	0.074(0.056, 0.092) <0.001	0.059 (0.042, 0.075) <0.001	0.007 (−0.013, 0.028) 0.493
	P for trend	<0.001	<0.001	0.501
Trochanter BMD	CMI	0.023 (0.016, 0.029) <0.001	0.015 (0.009, 0.021) <0.001	0.022 (0.008, 0.035) 0.002
	CMI Q1	Reference	Reference	Reference
	CMI Q2	0.042 (0.026, 0.058) <0.001	0.036(0.021, 0.051) <0.001	0.026 (0.011, 0.041) <0.001
	CMI Q3	0.062 (0.045, 0.080) <0.001	0.055(0.040, 0.071) <0.001	0.020 (0.003, 0.038) 0.022
	CMI Q4	0.100 (0.083, 0.116) <0.001	0.078(0.063, 0.093) <0.001	0.035(0.017, 0.054) <0.001
	P for trend	<0.001	<0.001	<0.001
Intertrochanter BMD	CMI	0.034 (0.024, 0.043) <0.001	0.022(0.014, 0.030) <0.001	0.034(0.015, 0.053) <0.001
	CMI Q1	Reference	Reference	Reference
	CMI Q2	0.046 (0.022, 0.070) <0.001	0.036 (0.016, 0.057) 0.001	0.011 (−0.011, 0.032) 0.325
	CMI Q3	0.096 (0.072, 0.121) <0.001	0.084 (0.063, 0.106) <0.001	0.027 (0.002, 0.051) 0.033
	CMI Q4	0.142 (0.118, 0.165) <0.001	0.109 (0.088, 0.130) <0.001	0.039 (0.014, 0.065) 0.003
	P for trend	<0.001	<0.001	0.002

Model 1: no covariates were adjusted.Model 2: age, gender and race were adjusted.Model 3: age, gender, race, BMI, hip circumference, poverty income ratio, smoking status, alcohol status, hypertension status, diabetes status, blood urea nitrogen, blood creatinine, globulin, blood total protein, uric acid, glycohemoglobin, serum phosphorus, serum calcium, serum iron, total cholesterol (TC), low-density lipoprotein cholesterol (LDL-C) were adjusted.

### Subgroup analysis

We performed subgroup analyses and interaction tests stratified by age, gender, race, BMI, diabetes status and hypertension status to evaluate whether the relationship between CMI and BMD is consistent in the general population, and the results are shown in Tables 3–8. When analyzed stratified by gender, the positive effect of CMI on BMD was predominantly seen in the female population (*p* < 0.05), particularly in Total femur BMD and Intertrochanter BMD (*p* < 0.01). When analyzed stratified by race, the positive correlation effect of CMI between total femoral BMD, trochanter BMD, and intertrochanter BMD was predominantly seen in non-Hispanic white people (*p* < 0.01). When analyzed stratified by age, the positive effect of CMI on total femoral BMD and Intertrochanter BMD was concentrated in people aged ≥60 years (*p* < 0.01). In contrast, the positive effect of CMI on trochanter BMD was concentrated in people aged <60 years. Furthermore, the positive correlation between CMI and BMD was significantly affected by age (P for interaction: 0.024). When analyzed stratified by BMI, the positive correlation of CMI on femoral BMD was mainly concentrated in people with BMI ≥30, particularly in Total femur BMD, Trochanter BMD and Intertrochanter BMD (*p* < 0.01). And the positive correlation of CMI on total femoral BMD and trochanter BMD was significantly influenced by the effect of BMI (P for interaction: 0.031; 0.043). When analyzed stratified by diabetes status, the positive correlation between CMI and total femur BMD, trochanter BMD, and intertrochanter BMD was predominantly observed in individuals with diabetes (*p* < 0.01). Moreover, the impact of CMI on trochanter BMD was significantly modified by diabetes status (P for interaction: 0.044). In the analysis stratified by hypertension status, the positive association of CMI with total femur BMD, trochanter BMD, and intertrochanter BMD was primarily evident in those with hypertension (*p* < 0.01). Additionally, the influence of CMI on total femur BMD and intertrochanter BMD was significantly modulated by hypertension status (P for interaction: 0.005; 0.010).

**Table 3:** Subgroup analysis of the association between CMI and BMD (stratified by gender).

		Adjust model β (95% CI), *p* value
	Stratified by gender	
Total femur BMD	Male	0.011 (−0.008, 0.031) 0.250
	Female	0.040 (0.014, 0.067) 0.003
	P for interaction	0.614
Femur neck BMD	Male	−0.022 (−0.042, −0.002) 0.028
	Female	0.029 (0.003, 0.055) 0.030
	P for interaction	0.101
Trochanter BMD	Male	0.015 (−0.004, 0.034) 0.116
	Female	0.026 (0.005, 0.048) 0.018
	P for interaction	0.686
Intertrochanter BMD	Male	0.019 (−0.005, 0.042) 0.122
	Female	0.044 (0.012, 0.077) 0.008
	P for interaction	0.796

Age, race, BMI, hip circumference, poverty income ratio, smoking status, alcohol status, hypertension status, diabetes status, blood urea nitrogen, blood creatinine, globulin, blood total protein, uric acid, glycohemoglobin, serum phosphorus, serum calcium, serum iron, total cholesterol (TC), low-density lipoprotein cholesterol (LDL-C) were adjusted.

**Table 4:** Subgroup analysis of the association between CMI and BMD (stratified by race).

		Adjust model β (95% CI), *p* value
	Stratified by race	
Total femur BMD	Mexican American	0.039 (−0.004, 0.082) 0.075
	Other Hispanic	−0.025 (−0.065, 0.014) 0.209
	Non-Hispanic White	0.036 (0.011, 0.062) 0.006
	Non-Hispanic Black	0.019 (−0.028, 0.065) 0.431
	Non-Hispanic Asian	0.076 (0.021, 0.131) 0.007
	Other Race—Including Multi-Racial	−0.071 (−0.188, 0.046) 0.247
	P for interaction	0.573
Femur neck BMD	Mexican American	0.015 (−0.028,0.058) 0.489
	Other Hispanic	−0.040 (−0.082, 0.002) 0.067
	Non-Hispanic White	0.007 (−0.018, 0.032) 0.591
	Non-Hispanic Black	−0.010(−0.055, 0.035) 0.666
	Non-Hispanic Asian	0.065 (0.009, 0.122) 0.024
	Other Race—Including Multi-Racial	−0.051(−0.188, 0.086) 0.470
	P for interaction	0.639
Trochanter BMD	Mexican American	0.023 (−0.016, 0.063) 0.253
	Other Hispanic	−0.020 (−0.056, 0.017) 0.292
	Non-Hispanic White	0.036 (0.014, 0.059) 0.002
	Non-Hispanic Black	0.006 (−0.035, 0.047) 0.770
	Non-Hispanic Asian	0.033 (−0.015, 0.080) 0.177
	Other Race—Including Multi-Racial	−0.096(−0.187, −0.004) 0.051
	P for interaction	0.310
Intertrochanter BMD	Mexican American	0.044 (−0.009, 0.098) 0.1058
	Other Hispanic	−0.026(−0.070, 0.018) 0.251
	Non-Hispanic White	0.043 (0.012, 0.075) 0.007
	Non-Hispanic Black	0.022 (−0.035, 0.078) 0.449
	Non-Hispanic Asian	0.116 (0.050, 0.182) 0.001
	Other Race—Including Multi-Racial	−0.070 (−0.214, 0.074) 0.353
	P for interaction	0.601

Age, gender, BMI, hip circumference, poverty income ratio, smoking status, alcohol status, hypertension status, diabetes status, blood urea nitrogen, blood creatinine, globulin, blood total protein, uric acid, glycohemoglobin, serum phosphorus, serum calcium, serum iron, total cholesterol (TC), low-density lipoprotein cholesterol (LDL-C) were adjusted.

**Table 5:** Subgroup analysis of the association between CMI and BMD (stratified by age).

		Adjust model β (95% CI), *p* value
	Stratified by age	
Total femur BMD	<60 year	0.017 (−0.009, 0.043) 0.192
	≥60	0.030 (0.011, 0.050) 0.003
	P for interaction	0.176
Femur neck BMD	<60 year	−0.008 (−0.036, 0.019) 0.541
	≥60	0.008 (−0.011, 0.027) 0.402
	P for interaction	0.587
Trochanter BMD	<60 year	0.031 (0.009, 0.053) 0.007
	≥60	0.016 (−0.002, 0.033) 0.080
	P for interaction	0.876
Intertrochanter BMD	<60 year	0.012 (−0.018, 0.043) 0.429
	≥60	0.046 (0.022, 0.071) <0.001
	P for interaction	0.024

Gender, race, BMI, hip circumference, poverty income ratio, smoking status, alcohol status, hypertension status, diabetes status, blood urea nitrogen, blood creatinine, globulin, blood total protein, uric acid, glycohemoglobin, serum phosphorus, serum calcium, serum iron, total cholesterol (TC), low-density lipoprotein cholesterol (LDL-C) were adjusted.

**Table 6:** Subgroup analysis of the association between CMI and BMD (stratified by BMI).

		Adjust model β (95% CI), *p* value
	Stratified by BMI	
Total femur BMD	<25	−0.006 (−0.046, 0.034) 0.775
	25–29.9	0.017 (−0.008, 0.042) 0.189
	≥30	0.055 (0.032, 0.078) <0.001
	P for interaction	0.031
Femur neck BMD	<25	−0.031(−0.069, 0.008) 0.117
	25–29.9	−0.006(−0.032, 0.019) 0.614
	≥30	0.024 (0.002, 0.047) 0.036
	P for interaction	0.137
Trochanter BMD	<25	−0.006 (−0.043, 0.031) 0.751
	25–29.9	0.018 (−0.004, 0.039) 0.109
	≥30	0.046 (0.026, 0.066) <0.001
	P for interaction	0.043
Intertrochanter BMD	<25	0.009 (−0.041, 0.059) 0.719
	25–29.9	0.022 (−0.009, 0.054) 0.161
	≥30	0.063 (0.036, 0.091) <0.001
	P for interaction	0.107

Age, gender, race, hip circumference, poverty income ratio, smoking status, alcohol status, hypertension status, diabetes status, blood urea nitrogen, blood creatinine, globulin, blood total protein, uric acid, glycohemoglobin, serum phosphorus, serum calcium, serum iron, total cholesterol (TC), low-density lipoprotein cholesterol (LDL-C) were adjusted.

**Table 7:** Subgroup analysis of the association between CMI and BMD (stratified by diabetes status).

		Adjust model β (95% CI), *p* value
	Stratified by diabetes status	
Total femur BMD	Yes	0.031 (0.013, 0.050) <0.001
	No	0.007 (−0.023, 0.036) 0.664
	P for interaction	0.241
Femur neck BMD	Yes	0.016 (−0.002, 0.035) 0.078
	No	−0.039 (−0.068, −0.011) 0.008
	P for interaction	0.012
Trochanter BMD	Yes	0.028 (0.012, 0.044) <0.001
	No	0.005 (−0.023, 0.032) 0.735
	P for interaction	0.044
Intertrochanter BMD	Yes	0.033 (0.011, 0.056) 0.003
	No	0.028 (−0.009, 0.066) 0.143
	P for interaction	0.803

Age, gender, race, hip circumference, poverty income ratio, smoking status, alcohol status, hypertension status, BMI, blood urea nitrogen, blood creatinine, globulin, blood total protein, uric acid, glycohemoglobin, serum phosphorus, serum calcium, serum iron, total cholesterol (TC), low-density lipoprotein cholesterol (LDL-C) were adjusted.

**Table 8:** Subgroup analysis of the association between CMI and BMD (stratified by hypertension status).

		Adjust model β (95% CI), *p* value
	Stratified by hypertension status	
Total femur BMD	Yes	0.057 (0.033, 0.081) <0.001
	No	0.015 (−0.005, 0.034) 0.151
	P for interaction	0.005
Femur neck BMD	Yes	−0.002 (−0.020, 0.019) 0.941
	No	0.010 (−0.014, 0.033) 0.434
	P for interaction	0.210
Trochanter BMD	Yes	0.038 (0.017, 0.059) <0.001
	No	0.018 (0.002, 0.036) 0.044
	P for interaction	0.167
Intertrochanter BMD	Yes	0.071 (0.041, 0.101) <0.001
	No	0.020 (−0.004, 0.044) 0.107
	P for interaction	0.010

Age, gender, race, hip circumference, poverty income ratio, smoking status, alcohol status, BMI, diabetes status, blood urea nitrogen, blood creatinine, globulin, blood total protein, uric acid, glycohemoglobin, serum phosphorus, serum calcium, serum iron, total cholesterol (TC), low-density lipoprotein cholesterol (LDL-C) were adjusted.

### Saturation effect analysis between CMI and BMD

As shown in Table 9 there was a saturation effect of CMI between total femoral BMD, trochanter BMD, and intertrochanter BMD with thresholds of 1.073, 1.431, and 1.073. There was a significant positive effect on BMD when CMI was below the threshold, and the regression coefficient decreased when CMI exceeded the threshold.

**Table 9:** Saturation effect analysis of CMI on BMD.

	Total femur BMD	Femur neck BMD	Trochanter BMD	Intertrochanter BMD
Fitting by the standard linear model	0.028 (0.020, 0.036) <0.001	0.001 (−0.014, 0.016) 0.9036	0.023 (0.016, 0.029) <0.001	0.034 (0.024, 0.043) <0.001
Fitting by the two-piecewise linear model				
Inflection point (k)	1.073	0.138	1.431	1.073
< K point effect 1	0.136 (0.113, 0.158) <0.001	1.093 (0.439, 1.747) 0.001	0.061 (0.032, 0.090) <0.001	0.163 (0.136, 0.190) <0.001
> K point effect 2	0.003 (−0.007, 0.012) 0.595	−0.004 (−0.019, 0.012) 0.623	0.001 (−0.007, 0.010) 0.725	0.002 (−0.009, 0.013) 0.686
Effect 2 minus effect1	−0.133 (−0.160, −0.107) <0.001	−1.097 (−1.754, −0.440) 0.001	−0.060 (−0.092, −0.027) <0.001	−0.161 (−0.193, −0.130) <0.001
Predicted value of the equation at the folding point	0.995 (0.981, 1.009)	0.746 (0.736, 0.757)	0.759 (0.742, 0.776)	1.186 (1.170, 1.203)
Log-likelihood ratio test	<0.001	<0.001	<0.001	<0.001

## Discussion

A total of 1,650 subjects, enrolled from 2017 to March 2020 (pre-pandemic period), were included in this study to evaluate the effect of CMI index on femur BMD. Our findings indicated that CMI had a significant positive correlation with total femoral BMD, trochanter BMD and intertrochanter BMD. Additionally, factors such as age, BMI, gender, diabetes, and hypertension status significantly influenced this correlation. However, there was no significant correlation between CMI and Femur neck BMD. This study is the first to identify a relationship between CMI and BMD, further demonstrating that BMD is closely related to fat metabolism.

CMI is influenced by a combination of triglycerides (TG), high-density lipoprotein cholesterol (HDL-C), height, and waist circumference. Specifically, an increase in TG levels, a decrease in HDL-C levels, an increase in height, and a decrease in waist circumference all contribute to an increase in CMI. While many previous studies have examined these variables individually, the relationship between HDL-C and BMD has been particularly controversial. For example, In a screening of 1,304 women, Zolfaroli et al. identified a positive correlation between HDL-C and bone mineral density in the lumbar spine and femoral neck (20). Conversely, Han et al. found in a case–control study of 710 people that those with osteoporosis had higher HDL-C (21). Similarly, Kim et al. found a negative correlation between HDL-C and BMD and the correlation was not affected by gender in a cross-sectional study of a Korean population (9). Furthermore, Cui’s study of 1,035 male and 3,953 female healthy volunteers found that Subjects have a significant reduction in bone mass when HDL-C is greater than 1.56 mmol/L (22). In a cross-sectional study of a larger population, Xie et al. found a U-shaped relationship between HDL-C and lumbar spine BMD, with a negative correlation between HDL-C and lumbar spine BMD when HDL-C was <0.98 (mmol/L), and a positive correlation when this value was exceeded (23). Supporting these findings, cellular studies have also confirmed this relationship. Huang et al. found that HDL-C promotes cholesterol efflux from osteoblasts by upregulating ABCG1 expression, which disrupts the dynamic balance of cholesterol in osteoblasts, thereby inducing apoptosis and impairing osteoblast formation (24). Kha et al. found that high levels of HDL-C inhibited bone differentiation, which is mainly related to the fact that HDL-C removes oxidized sterols from the peripheral circulation which play an important role in osteogenic differentiation (25). In this study, the conversion of high HDL-C to CMI is reflected as low CMI, and the population in the low CMI group (Q1) in this study had lower BMD than the high CMI population, which briefly demonstrates the negative correlation between HDL-C and BMD.

I Furthermore, numerous studies have also explored the relationship between TG and BMD. Xu et al. found that TG can be used as a diagnostic indicator to assist in the diagnosis of osteoporosis in older women, and that TG levels were higher in the osteoporosis group than in the normal population (26). Similarly, Wang et al. analyzed NHANES data from 2017–2020 and found a positive correlation between TG and lumbar spine BMD at TG > 2.597 mmoL/L in the older age group of 50 years or older (18). These findings suggest that TG plays a significant role in bone health, and when TG was included in the calculation of CMI, it demonstrated a significant positive correlation between CMI and BMD.

Other indicators of lipid metabolism are also strongly associated with BMD. For example, there is a negative correlation between low-density lipoprotein cholesterol (LDL-C) and BMD, which is likely due to the fact that high levels of LDL-C promote osteoclastogenesis. Elevated LDL-C levels are believed to contribute to the loss of bone mineral density by enhancing osteoclast activity (27). This process is closely linked to key enzymes in the cholesterol metabolic pathway, such as HMG-CoA reductase.

The primary mechanisms underlying these effects include the removal of oxidized sterols from peripheral tissues by HDL-C and the inhibition of osteoblast differentiation by oxidation products of LDL-C. Cholesterol, high-density lipoprotein cholesterol (HDL-C) and low-density lipoprotein cholesterol (LDL-C) induced progenitor MSCs (mesenchymal stem cells) to undergo lipogenic, rather than osteogenic differentiation and induced RANKL—(nuclear factor receptor activator of κB ligand-) dependent osteoclast differentiation (25, 28). Trimpou et al. further observed necrosis of the femoral head under electron microscopy, noting a significant increase in both the number and size of adipocytes, which suggests that lipid metabolism may play a crucial role in the formation of bone geometry (29). Meanwhile, fatty acids, phospholipids and several endogenous metabolites have been reported to play a key role in the homeostatic level of bone. These molecules influence the survival and function of osteoblasts, participate in the bone mineralization process, and even regulate various critical signaling pathways (30).

Moreover, CMI is a new obesity index that reflects the distribution and functional status of visceral fat in the body. It represents both the degree of obesity and an individual’s lipid levels (17). Abdominal obesity is characterized by an increase in visceral fat, mainly in the form of an increased BMI. The relationship between BMI and BMD is currently controversial, and Asuman found that BMD was significantly higher in overweight individuals than in normal weight individuals (31). A cross-sectional study by Ma et al., based on NHANES data from 2005–2018, observed that the relationship between BMI and BMD is not simply linear; instead, there are saturation points where optimal BMD can be achieved by maintaining a slightly higher BMI (32). JIA et al. found that the lower the BMI, the greater the BMD loss in 128 postmenopausal women with osteoporotic fractures (33). However, Auslader et al. found no significant correlation between BMI and BMD in young women (34). The susceptibility of the female population to BMD is closely related to the decrease in oestrogen levels and the increase in osteoclast activity in postmenopausal women, resulting in a disruption of the balance between accelerated bone resorption and bone remodeling in favour of bone resorption (5, 35). This study also confirmed the significant effect of CMI on total femur BMD and trochanter BMD, with BMI playing a role in these outcomes. Additionally, our stratified analysis revealed that female BMD is more susceptible to the influence of CMI.

Furthermore, recent studies have demonstrated that the cardiometabolic index (CMI) is strongly associated with metabolic diseases such as cardiovascular disease, diabetes, and insulin resistance (36–38). Previous studies have shown that metabolic diseases such as diabetes mellitus, insulin resistance, and non-alcoholic fatty liver disease are strongly associated with BMD (13–15, 39). The haemodynamic changes in bone due to cardiovascular diseases such as hypertension and the hyperinsulinism associated with diabetes mellitus and insulin resistance will both promote osteoblast differentiation leading to increased BMD (40, 41). In addition, insulin resistance is often accompanied by changes in the secretion of adipokines, which may regulate bone metabolism through the RANKL/RANK/OPG system, promoting bone formation and leading to increased BMD (42) Consistent with these findings, a recent study demonstrated a positive relationship between insulin resistance, dysglycemia and BMD in young Indian women (39). This study also identified a positive correlation between CMI and BMD, particularly in hypertensive and diabetic populations, as revealed through stratified analyses.

In conclusion, osteoporosis and lipid metabolism are closely related. Therefore, the cardiometabolic index (CMI) can be used as a novel indicator of lipid metabolism levels in the body, which may aid in the diagnosis and prevention of osteoporosis. The strengths of this study include the use of a complex multi-stage probability sampling design, which enhances the reliability and representativeness of the findings. However, there are several limitations to consider. First, this study employed a primarily cross-sectional design, which limits the ability to establish a causal relationship between CMI, BMD, and osteoporosis. Additionally, bone microarchitecture and turnover were not evaluated, therefore limiting our ability to definitively determine the impact of increased CMI on bone health dynamics. Future research should include these parameters to provide a more comprehensive understanding of the relationship between CMI and bone health. Finally, the covariates included in this study were limited; however, the observed correlation between CMI and osteoporosis remains sufficiently robust, making it unlikely to be significantly affected by unmeasured factors.

## Conclusion

This study revealed that total femur BMD, trochanter BMD, and intertrochanter BMD increase with higher CMI levels. This correlation was more pronounced in individuals aged 60 years and older, with a BMI of 30 or greater, and in those with diabetes or hypertension. However, further large-scale prospective studies are needed to validate these findings.

## Data Availability

Publicly available datasets were analyzed in this study. The data of this study are publicly available on the NHANES datasets. Specific data on this study is detailed at the following link: https://wwwn.cdc.gov/nchs/nhanes/continuousnhanes/default.aspx?Cycle=2017-2020.
